# To the Editors of the Medical and Physical Journal

**Published:** 1813-09

**Authors:** 


*To the Editors of the Medical and Physical Journal.*

GENTLEMEN,

*To the Editors of the Medical and Physical Journal.*

GENTLEMEN,

AS the London Committee of Apothecaries intend to renew their application to Parliament in the next session, for a bill to regulate the profession, and earnestly solicit the continued co-operation of the country practitioners in furtherance of the same object, it behoves the latter gentlemen to re-consider the subject, and estimate as correctly as possible all the probable consequences of regulating and restraining the practice of the profession by such legislative provisions, or by any similar ones, as those last proposed by the London Committee.

There was no doubt but that the majority of the profession would hail with approbation any proposal to improve its usefulness and respectability, particularly when coming from such a source as it did; but it is to be feared that this sentiment, and the expectations which were raised, have been succeeded by some degree of disappointment; and some change of opinion, as to the possibility of making any law on the subject that would be equally advantageous to the public and the profession, and whether it would not be productive of more trouble and vexation than utility.

 There

There is, I presume, no doubt that there is less need of such a law now than at any former time, from the rapid and important improvements that have taken place in our profession, which has gone on at least with equal if not with greater advances than any other of the liberal arts; and which, I believe, has received, and does still receive, a full and ample share of public estimation. I would beg permission to recal the attention of the country practitioners particularly to this subject, and submit to them whether they are not of opinion that they have some reason to decline their renewal of support to a measure which is not likely to be productive of benefit to the public or to the profession?

I am, Gentlemen, your's,

*July* 18, 1813. SURRIENSIS.

